# Supplementary material for: The Use of Thromboelastography in Percutaneous Coronary Intervention and Acute Coronary Syndrome in East Asia: A Systematic Literature Review
Source: J Clin Med. 2022 Jun 24;11(13):3652. doi: 10.3390/jcm11133652 (PMC9267871; doi:10.3390/jcm11133652)
Supplement: Supplementary file 1 [file jcm-11-03652-s001.zip › jcm-1648898-supplementary.pdf]

The use of thromboelastography in percutaneous coronary intervention and acute coronary syndrome in East Asia: A systematic literature review

Authors: Ou Xu[1], Jan Hartmann[1], Tang Yi-Da[2], Joao Dias[1]

Supplementary material

Table S1: Search terms

Interventional cardiology terms	Drug treatment of cardiovascular disease terms	Countries when investigators are bases	TEG technology terms	Filters
percutaneous coronary intervention<br>OR<br>PCI<br>OR<br>transcatheter aortic valve replacement<br>OR<br>transcatheter aortic valve implant*<br>OR<br>transcatheter aortic valve intervent*<br>OR<br>TAVR<br>OR<br>TAVI<br>OR<br>electrophysiol*	[arrhythmia*<br>OR<br>acute coronary syndrome*<br>OR<br>coronary artery disease<br>OR<br>heart<br>OR<br>myocardial<br>OR<br>coronary<br>OR<br>cardiac<br>OR<br>cardiovascular]<br>AND<br>[platelet aggregation inhibitors<br>OR<br>antiplatelet<br>OR<br>anticoagulant<br>OR<br>antithrombo*]	China<br>OR<br>Japan<br>OR<br>Korea<br>OR<br>Thailand<br>OR<br>Singapore<br>OR<br>Hong Kong<br>OR<br>Malaysia<br>OR<br>Indonesia<br>OR<br>Philippines<br>OR<br>Myanmar<br>OR<br>Laos<br>OR<br>Cambodia<br>OR<br>Vietnam<br>OR<br>Taiwan	Thromboelasto*<br>OR<br>TEG<br>OR<br>Thrombelasto*<br>OR<br>PlateletMapping	Published in the last 10 years<br>AND<br>[English language<br>OR<br>Chinese language]

Table S2: SIGN grading

Article	Study Type	SIGN Rating*
**PubMed: English language studies**		
Yan, XQ, et al. Ann Palliat Med 2021. 10(3):2448-2457	Non-randomized prospective study	2+ or 2-
Gorog, DA, et al. Cardiovasc Res 2021. 117(2): 623-634	RCT	1-
Wu, HY et al. Acta Pharmacol Sin 2020. 41(2): 192-197	Post hoc analysis of prospective observational study	2+ or 2-
Wei, W et al. J Thorac Dis 2020. 12(10): 5811-5821	Non-randomized observational study	2+
Park, Y et al. J Clin Med 2020. 9(6): 1678	RCT	1+
Cheng, D et al. Biosci Rep 2020. 40(7)	Non-randomized prospective study	2+ or 2-
Peng, W et al. Cardiovasc Ther 2019.	Non-randomized prospective study	2-
Li, X et al. Front Pharmacol 2019. 10: 1158	Non-randomized prospective study	2-
Li, X et al. Basic Clin Pharmacol Toxicol 2019. 124(1): 84-93	Non-randomized prospective study	2-
Cao, B et al. Aging (Albany NY) 2019. 11(20): 8925-8936	RCT	2-
Zhuo, ZL et al. Anatol J Cardiol 2018. 19(2): 123-129	Non-randomized prospective study	2-
Yang, B et al. Iran J Public Health 2018. 47(7): 952-957	RCT	1+
Tang, YD et al. Circulation 2018. 137(21): 2231-2245	RCT	1+
Hou, X et al. J Clin Lab Anal 2018. 32(5): e22369	Non-randomized prospective study	2+
Gong, W et al. J Am Heart Assoc 2018. 7(15): e008808	Non-randomized prospective study	2+
Zhu, P et al. Chin Med J (Engl) 2017. 130: 2899-2905	Non-randomized prospective study	2+
Nie, XY et al. J Zhejiang Univ Sci B 2017. 18(1): 37-47	Non-randomized prospective study	2-
Fu, DL et al. Chin Med J (Engl) 2017. 130(16): 1914-1918	Non-randomized prospective study	2-
Yao, Y et al. Chin Med J (Engl) 2016. 129(19): 2269-2274	Non-randomized prospective study	2-
Li, DD et al. J Geriatr Cardiol 2016. 13(4): 282-289	Non-randomized prospective study	2-

Dong, P et al. Med Sci Monit 2016. 22: 4929-4936	RCT	2-
Wu, ZK et al. J Geriatr Cardiol 2015. 12(4): 378-382	Non-randomized prospective study	2+
Tang, XF et al. Chin Med J (Engl) 2015. 128(6): 774-779	Non-randomized prospective study	2+
Sun, B et al. Genet Mol Res 2015. 14(1): 1434-1442	Non-randomized prospective study	2-
Liu, J et al. Chin Med J (Engl) 2015. 128(16): 2183-2188	Non-randomized prospective study	2-
Zhu, P et al. Platelets 2021. 32(5):684-689	Non-randomized prospective study	2+
Zhang, M et al. Eur Rev Med Pharmacol Sci 2020. 24(20): 10753-10768	RCT	1
Zhu, P et al. Platelets 2019. 30(7): 901-907	Non-randomized prospective study	2-
Koh, JS et al. Thromb Haemost 2019. 119(2): 264-273	RCT	1
Zhang, S et al. Cardiology 2018. 140(1): 21-29	Non-randomized prospective study	1
Chen, Y et al. Cardiovasc J Afr 2018. 29(6): 357-361	Non-randomized prospective cross-sectional study	2+
Song, Y et al. Biomed Environ Sci 2017. 30(12): 898-906	Non-randomized prospective study	2+
Koh, JS et al. Platelets 2017. 28(2): 187-193	RCT	1+
Yao, Y et al. Thromb Res 2016. 141: 28-34	Non-randomized prospective study	2-
Tang, N et al. Scand J Clin Lab Invest 2015. 75(3): 223-229	Non-randomized prospective study	2+
Tian, KP et al. Nan Fang Yi Ke Da Xue Xue Bao 2016. 37(4): 533-536	Non-randomized prospective study	2-
Xia, JG et al. Beijing Da Xue Xue Bao Yi Xue Ban 2015. 47(3): 494-498	RCT	1
Zhong, T et al. Zhongguo Shi Yan Xue Ye Xue Za Zhi 2018. 26(5): 1484-1491	Cross sectional case control study	2
Luo, Y et al. Zhonghua Wei Zhong Bing Ji Jiu Yi Xue 2020. 32(8): 994-997	RCT	1
Zhang, M et al. Perfusion 2020.	RCT	2+

Xu, XR et al. Zhonghua Nei Ke Za Zhi 2016. 55(12): 932-936	RCT	1-
Xu, JJ et al. Zhonghua Xin Xue Guan Bing Za Zhi 2017. 45(2): 116-120	Non-randomized prospective study	2+
**Chinese Databases: Chinese language studies**		
Li, B. Clinical Medicine 2020. 0(1): 57-59	RCT	1+
Li, D et al. Chinese Clinical Journal of Thoracic and Cardiovascular Surgery 2019. 26(2): 137-141	Cohort study	2-
Wu, H et al. Shanghai medical journal 2018. 41(1): 9-13	Case control study	2+
Cui, Y et al. Chinese Cardiovascular Disease Research 2018. 16(1): 34-38	Cohort study	2-
Dana, W et al. Journal of Clinical Cardiovascular Diseases 2018. 34(7): 685-690	Cohort study	2-
Chen, Z. Northern Pharmacy 2017. 14(9): 23-23	Non-randomized prospective study	2-
Wang, J. Clinical Medicine and Research 2018. 0(66): 64-64	Cohort study	2-
Cui, Y et al. Journal of Integrated Cardiovascular and Cerebrovascular Diseases 2018. 16(4): 461-463	Case control study	2+
Li, G et al. Journal of Clinical Blood Transfusion and Testing 2017. 19(1): 59-63	Case control study	2+
Miao, L et al. Journal of Clinical Cardiovascular Diseases 2016. 32(4): 334-338	RCT	1+ or 1-
Ma, Y et al. Modern Applied Pharmacy in China 2017. 34(4): 587-590	Non-randomized prospective study	2-
Chen, ML et al. Journal of Diagnostic Theory and Practice 2016. 0(2):142-147	Non-randomized prospective study	2-
Wu, Y et al. Journal of Clinical Hematology (Blood Transfusion and Testing) 2016. 29(1): 120-122	Retrospective study	2-

World Chinese Association of laboratory and pathologists; Laboratory physicians branch of Chinese Medical Association; Professional Committee of cardiovascular Laboratory Medicine. Journal of Clinical Medical Research and Practice 2018. 3(19): 201-201	Consensus	4
Wei, H et al. Chinese Journal of Emergency Resuscitation and Disaster Medicine 2018. 13(10): 946-948	RCT	1-
Liang, D et al. Clinical Medicine and Research 2016. 0(58): 186-187	Cohort study	2-
Huang, L et al. Chinese Journal of Geriatric Multi-organ Diseases 2017. 16(11): 846-849	RCT	1-
Hodgs et al. Clinical Medicine and Research 2020. Volume(24): 34-35	RCT	1+
Cui, H. New Word Journal of Diabetes 2019. 22(14): 22-23	Case control study	2-
Ren, D et al. Journal of Cardiovascular and Vascular Diseases 2019. 38(7): 725-730	Case control study	2+
He, W. Modern Medicine and Health 2019. 35(S01): 27-28	Case control study	2-
Liu, W et al. Journal of Modern Integrative Medicine 2017. 26(22): 2487-2489	Historical comparison	2-
Zhao, Y et al. Journal of Laboratory Medicine and Clinical 2017. 28(3): 37-40	Non-randomized prospective study	2-
Chen, Q. Thrombosis and hemostasis 2016. 22(6): 611-613	RCT	1-
Zhang, Q et al. Chinese Journal of Pharmaceutical Science 2016. 6(11): 97-99	RCT	1-
Yanjun, Y et al. Chinese Journal of General Medicine 2016. 14(8): 1289-1292	Non-randomized prospective study	2+

Zhang, Y et al. Jilin Medicine 2020. 41(7): 1587-1589	RCT	1-
Zhang, Q et al. Clinical Research Journal 2019. 27(11): 19-21	Non-randomized prospective study	2+
Liu, W et al. Lingnan Journal of Cardiovascular Diseases 2019. 25(1): 53-57	Non-randomized prospective study	2+
Wu, D et al. Chinese Journal of Hospital Pharmacy 2018. 38(7): 759-762	Non-randomized prospective study	2-
Wu, F. Journal of Contemporary Medicine 2018. 16(24): 25-26	RCT	1-
Miao, L et al. Chinese Journal of Clinical Laboratory Science 2017. 35(6): 439-443	Non-randomized prospective study	2+
Zhuang, J et al. Chinese Cardiovascular Disease Research 2017. 15(5): 445-450	RCT	1+
Huang, M et al. Journal of Practical Clinical Medicine 2017. 21(12): 49-52	RCT	1-
Li, H. Chinese Practical Medical Journal 2016. 43(22): 40-42	Non-randomized prospective	2-
Wén, TQ et al. China Medical Guide 2016. 14(4): 165-166	Retrospective case control study	2-
Fu, X. Journal of Medical Dietary Therapy and Health 2018. 0(12): 45-45	Retrospective case control study	2-
Li, X et al. Medical Journal of Chinese People’s Armed Police Forces 2018. 29(1): 14-16	RCT	1-
Jūn, HY et al. Journal of Integrated Cardiovascular and Cerebrovascular Diseases 2017. 15(3): 358-360	Non-randomized prospective study	2+
Ma, J et al. Journal of Cardiovascular Diseases of Integrated Traditional Chinese and Western Medicine 2016. 4(24): 29-29	Non-randomized prospective study	2-
Cai, H. Knowledge of Cardiovascular Disease	Non-randomized prospective study	2+

Prevention and Treatment 2020. 10(22): 32-34		
Lin, G et al. Thrombosis and Hemostasis 2019. 25(2): 199-201	RCT	1-
Zhan, X et al. Chinese Journal of Evidence-based Cardiovascular Medicine 2017. 9(9): 1108-1111	RCT	1-
Xu, Y et al. Modern Practical Medicine 2016. 28(10): 1323-1324	Retrospective case control study	2+
Wei, H et al. Journal of Hunan University of Traditional Chinese Medicine 2016. 36(A02): 672-673	RCT	1-
Pan, B et al. Modern Medicine 2019. 47(9): 113-1138	Non-randomized prospective study	2-
Wu, D et al. Journal of Integrated Cardiovascular and Cerebrovascular Diseases 2019. 17(8): 1210-1212	Retrospective case control study	2-
Shen, W et al. Journal of Pla Medical College 2018. 39(2): 106-109	RCT	1+
Lin, T et al. Chinese Journal of Gerontology 2018. 38(7): 1568-1569	RCT	1-
Ma, C et al. Journal of Nanjing Medical University 2018. 38(10): 1415-1420	RCT	1-
Hou, X et al. Chinese Cardiovascular Disease Research 2016. 14(12): 1081-1085	RCT	1-
Chen, Z et al. Journal of Clinical Research 2016. 33(11): 2207-2209	Non-randomized prospective study	2-
Hao, Z. Capital Medical Journal 2020. 27(19): 41-42	Non-randomized prospective study	2-
Brief News. Journal of Military Surgeon in Southwest China 2019. 21(6): 501-504	Non-randomized prospective study	2+

Du, Y et al. China Medical Herald Journal 2019. 16(11): 50-53	Non-randomized prospective study	2-
Huang, X et al. Anhui Medical Journal 2017. 38(12): 1580-1582	Non-randomized prospective study	2-
Zhang, D et al. Chinese Journal of Modern Medicine 2017. 19(7): 17-20	Case control study	2-
Yan, F et al. Journal of Practical Drugs and Clinics 2017. 20(4): 423-428	Non-randomized prospective study	2-
Hu, Z et al. Beijing Medical Journal 2018. 40(9): 818-820	Non-randomized prospective study	2+
Li, A et al. Chinese Journal of Modern Medicine 2017. 27(1): 94-98	Non-randomized prospective study	2-
Bai, Y et al. Chinese Journal of Evidence-based Cardiovascular Medicine 2016. 8(12): 1491-1493	RCT	1-
Pei, Y et al. Journal of Clinical Psychosomatic Diseases 2020. 26(3): 45-47	RCT	1-
Liu, X et al. Journal of Clinical Hematology (Transfusion and Testing) 2020. 33(3): 425-427	Non-randomized prospective study	2-
Qiu, Y et al. Journal of Practical Medical Techniques 2020. 27(3): 284-286	RCT	1+
Wang, G et al. Journal of Community Medicine 2020. 0(4): 264-268	Case control study	2+
Zhu, J et al. Journal of Clinical Medicine Literature 2020. 7(96): 166-166	RCT	1-
Zhang, C et al. Medical Innovation in China 2018. 15(16): 27-30	Case control study	2-
Sun, A et al. Journal of Clinical Cardiovascular Diseases 2017. 33(5): 431-434	RCT	1+
Chen, W et al. Medical New Knowledge 2016. 26(5): 338-341	Cohort study	2-

Zhang, X et al. Shandong Medicine 2016. 56(5): 39-41	RCT	1-
Chen, Z et al. Shanxi Medical Journal 2020. 49(14): 1846-1848	Retrospective case control study	2-

*In order of increasing bias, SIGN rating describes the levels of evidence ranked 1–4. Study type and quality of data are used to produce four grades of recommendation defined as follows; 1++: high quality meta-analyses, systematic reviews of RCTs, or RCTS with very low risk of bias; 1+: well-conducted meta-analyses, systematic reviews, or RCTs with a low risk of bias; 1-: meta-analyses, systematic reviews, or RCTs with a high risk of bias; 2++: high quality systematic reviews of case control or cohort or studies, high quality case control or cohort studies with a very low risk of confounding or bias and a high probability that the relationship is causal; 2+: well conducted case control or cohort studies with a low risk of confounding or bias and a moderate probability that the relationship is causal; 2-: case control or cohort studies with a high risk of confounding or bias and a significant risk that the relationship is not causal; 3: non-analytic studies, e.g., case reports, case series; 4: expert opinion

SIGN, Scottish intercollegiate guidelines network; RCT, randomized controlled trial
